# Retraction: LncRNA SNHG3 enhances the malignant progress of glioma through silencing KLF2 and p21

**DOI:** 10.1042/BSR-2018-0420_RET

**Published:** 2021-10-14

**Keywords:** KLF2, p21, SNHG3

This Retraction follows an Expression of Concern relating to this article previously published by Portland Press.

This article is being retracted from *Bioscience Reports* at the request of the Editor-in-Chief and the Editorial Board following receipt of a notification from a reader, alerting the Editorial Board a duplicated image in Figure 2C as well as concerns with the similarity of data in Figure 3E and concerns with the Western blots in Figure 6C.

After notification by the Editorial Office, the authors agreed to the Retraction. Given the extent of the issues raised, the Editorial Board stand by the decision to retract the article.

